# An asymptomatic right atrial intramyocardial lipoma: a management dilemma

**DOI:** 10.1186/s12957-015-0441-9

**Published:** 2015-02-06

**Authors:** Haiyong Wang, Jiangwei Hu, Xiaolin Sun, Pingshan Wang, Zhenzong Du

**Affiliations:** Department of Cardiothoracic Surgery, Affiliated Hospital of Guilin Medical University, Guilin, 541001 China

**Keywords:** Heart neoplasm, Diagnosis, Surgery, Lipoma

## Abstract

**Background:**

The atrial intramyocardial lipomas are rare benign unusual tumors of the heart. The indication and best form of treatment for cardiac lipomas remain controversial.

**Case presentation:**

The atrial intramyocardial lipomas are rare benign unusual tumors of the heart. We report a 55-year-old Chinese female with a history of hypertension. Echocardiography and 64-slice computed tomography showed a fatty mass in the right atrium. Although she was asymptomatic, a surgical resection was indicated since the lipoma could cause an embolism and arrhythmias and its potential to enlarge. Surgery revealed an intramyocardial lipoma on the atrial free wall which was confirmed by histopathology. The patient remained asymptomatic after surgery, and no recurrence was seen after 1 year.

**Conclusions:**

Although cardiac lipomas are usually benign, tumor embolism, potential to enlarge, or intracardiac obstruction can cause a critical situation. Therefore, a surgical resection was indicated even in asymptomatic patients.

**Electronic supplementary material:**

The online version of this article (doi:10.1186/s12957-015-0441-9) contains supplementary material, which is available to authorized users.

## Background

Cardiac lipomas are relatively rare [1]. These well-encapsulated tumors are typically composed of mature fat cells. Most of them do not cause clinical symptoms so that they are found accidentally in the majority of cases. However, intramyocardial lipomas may cause changes in electrical conduction and supraventricular rhythm disturbances and may result in sudden death [2]. The indication and best form of treatment for cardiac lipomas remain controversial.

## Case presentation

**Figure 1:**
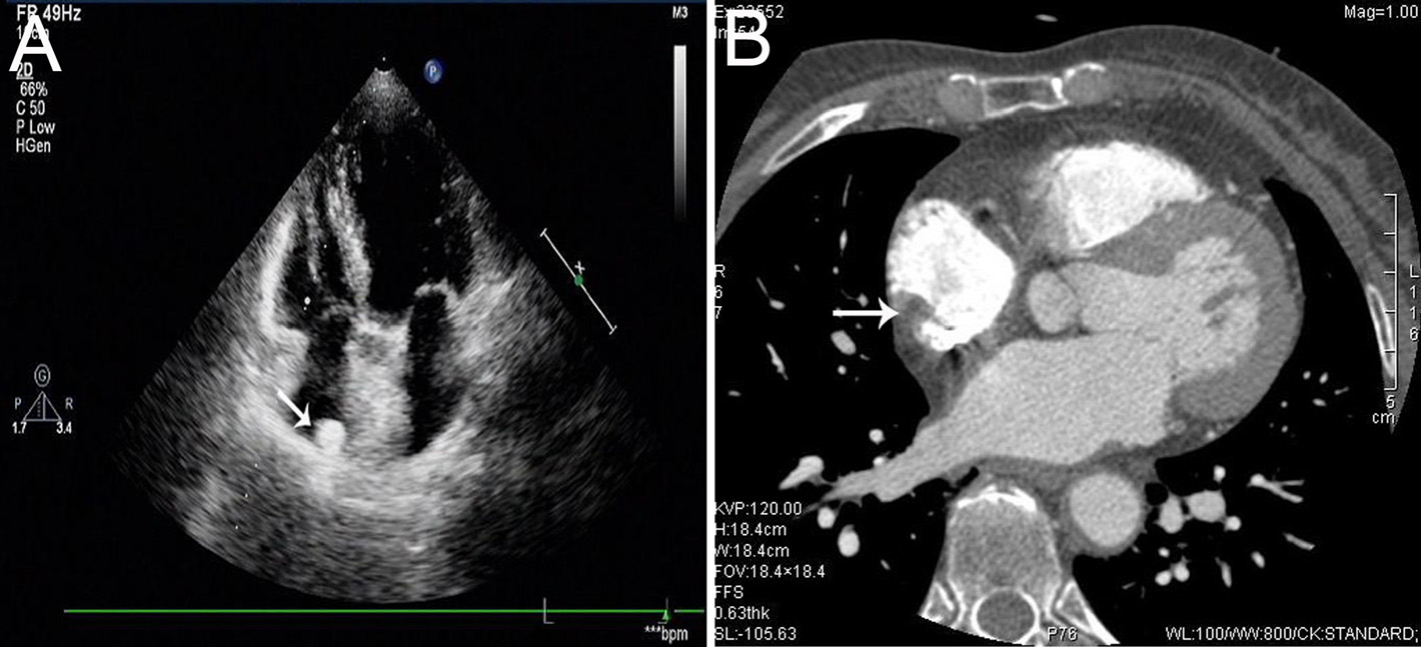
**Echocardiogram and computed tomography. (A)** Transthoracic echocardiogram showing a mass (arrow head) attached to the free wall of the right atrium. **(B)** A 64-multislice computed tomography (CT) showing a lipomatous mass (arrow head) on the free wall of the right atrium (arrow head).

A 55-year-old Chinese female with a history of hypertension was evaluated by the cardiologist. She had a normal pulse rate and a regular rhythm, and her blood pressure was 150/85 mmHg. No murmur or thrill was present. The chest radiograph was normal. Echocardiogram showed a mass in the right atrial free wall, measuring around 30 mm × 15 mm in diameter (Figure 1A). She was admitted to our hospital for further evaluation.

To obtain better definition of the mass, a 64-slice computed tomography (CT) was performed. An evaluation by means of CT demonstrated that the mass, which contained several thin septations, was consistent with lipoma. The mass occupied a part of the right atrial free wall (Figure 1B). But, we were uncertain whether the fatty tumor was epicardial, intramural, or endocardial.

**Figure 2:**
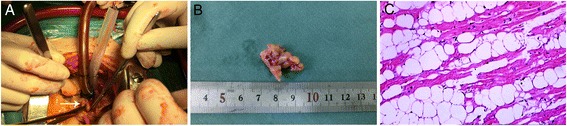
**Surgical specimens. (A)** Lipoma (arrow head) attached to the free wall of the right atrium—intraoperative view. **(B)** Tumor measured 32 mm × 17 mm × 7 mm. **(C)** Histological specimen shows a benign tumor composed of mature adipocytes lined with entrapped cardiac myocytes.

Surgical treatment was indicated. After routine median sternotomy, the patient was started on moderate systemic hypothermic (34°C) cardiopulmonary bypass with cannulation of the aorta ascendens and both venae cavae. The superior vena cava cannula was inserted very high in the superior vena cava so as to be away from the tumor. The inferior vena cava cannula was also inserted very low near the diaphragm. To reduce the risk of complications, the patients underwent open heart operation on beating hearts. After the right atrium was opened, there was a large, yellowish rubbery mass (32 mm × 17 mm × 7 mm) constituting part of the free wall of the right atrium (Figure 2A). After total tumor excision, the defect of atrial free wall was closed directly without any patch.

Gross examination revealed a 32 mm × 17 mm × 7 mm mass comprised predominantly of mature adipose tissue of an intramyocardial lipoma (Figure 2B). Histologically, the excised specimen showed many fatty structures lined by myocardium, suggesting that the tumor had indeed originated within the atrial wall (Figure 2C).

The patient recovered well on the ward and was discharged 8 days after the operation. On 1-year follow-up, the patient remained asymptomatic, with good clinical and echocardiography evaluations.

## Discussion

Primary cardiac tumors are very rare. Their incidence at autopsy ranges from 0.001% to 0.03%. Cardiac lipomas were first described by Albers in 1856 and are responsible for about 8% of the primary cardiac tumors [3,4]. Lipomas commonly occur in middle-aged and older adults. Most of them are subendocardial or epicardial, and only 25% are found in the myocardium [5]. The most frequent location is the left ventricle and the right atrium [6].

The clinical symptoms of lipomas are non-specific or absent and usually related to the location and size of the mass. Most often, they are discovered incidentally at echocardiography. These lesions can become symptomatic as a result of conduction disturbances, atrial arrhythmias, valvular dysfunction, and obstructive symptoms.

Modern imaging techniques such as echocardiography, multislice CT, and magnetic resonance imaging (MRI) all can provide reliable noninvasive diagnosis in relation to the location of the cardiac tumor [6,7]. However, as tissue characterization may not be possible by echocardiography, CT and MRI were suggestive of a well-defined space-occupying lesion adjacent to the cardiac border, suggesting the possibility of a cardiac tumor. Multislice computed tomography and cardiac magnetic resonance imaging in conjunction with fat saturation techniques are highly sensitive and specific techniques to diagnose cardiac lipomatous lesions and help to differentiate cardiac lipomas from other intracardiac masses. Lipomas usually have low density, which ranges from 80 to 115 Hounsfield units [8]. The spatial resolution of MRI can provide detailed information about the size and location of the tumor. By evaluating T1 and T2 characteristics of the tissue, as well as the ability to use fat suppression techniques in addition to gadolinium, the histopathologic characteristics of the tissue can be evaluated [9]. But in our case, without histopathological examination, it was impossible to identify the microscopic structure of the mass to determine the management and prognosis.

The management of cardiac tumors should be quick and accurate because they can progress silently and cause arrhythmias, cardiac compression, and valvular dysfunction [10]. Cardiac lipomas are so rare that no therapeutic guidelines have been established for the surgical indications of such cases. This creates therapeutic dilemmas, especially when the patient is asymptomatic. Some authors suggest that asymptomatic cardiac lipomas detected by chance should not be surgically corrected and that surgery be reserved only for patients with intractable arrhythmias, heart failure, thromboembolic sequelae, or inability to exclude liposarcomas [11].

Although it is a benign tumor remaining asymptomatic in many patients, it may cause a variety of complications, including compression of the coronary arteries or pericardial space (subepicardial), arrhythmias (intramyocardial), or outflow obstruction (subendocardial). Taking into account the fatal courses which have been reported for untreated symptomatic lipomas [12], surgical resection is the treatment of choice and in many cases leads to complete cure [13]. In order to reduce the morbidity of heart operations, we performed tumor resection on beating heart.

We resected the whole tumor in our case, without any patch for reconstruction of the free wall. If complete excision of the large lipoma is planned, reconstruction of the interatrial septum and cavity closure with autologous pericardium or Dacron must be planned [14]. Also, complete surgical resection should not be attempted if it will compromise vital structures, taking into consideration its rare malignant transformation and absence of recurrence after excision.

The first report of success in resection of epicardial lipoma occurred in 1954 by Maurer [12]. Nowadays, the safety of cardiopulmonary bypass and cardiac surgery techniques has made possible the safe surgical removal of these tumors. Prompt surgical excision of cardiac tumors often results in a complete cure, affording these patients an excellent long-term prognosis [15]. Moreover, the minimally invasive approach for resection of benign cardiac masses is a low-risk procedure and superior to standard full sternotomy. But the minimally invasive approach for the resection of benign cardiac tumors is limited and based only on few retrospective studies [16].

## Conclusions

Although cardiac lipomas are usually benign, tumor embolism, potential to enlarge, or intracardiac obstruction can cause a critical situation. Therefore, a surgical resection was indicated even in asymptomatic patients.

## Consent

Written informed consent was obtained from the patient for publication of this paper and accompanying images. A copy of the written consent is available for review by the Editor-in-Chief of this journal.
